# The Influence of Signaling on the Disfluency Effect in Multimedia Learning

**DOI:** 10.3389/fpsyg.2021.755804

**Published:** 2021-11-02

**Authors:** Tingming Lai, Jinkun Zhang

**Affiliations:** School of Psychology, Fujian Normal University, Fuzhou, China

**Keywords:** perceptual fluency, disfluency effect, signaling, multimedia learning, eye movement

## Abstract

Do students learn better with texts that are *slightly harder-to-read* (i.e., disfluent)? Previous research has yielded conflicting findings. The present study identified the boundary condition that determines when disfluent texts benefit learning. We used eye-tracking to examine the joint influence of text legibility (fluent vs. disfluent) and signaling (signaling vs. non-signaling) on multimedia learning. The results revealed that both disfluent text and signaling led to better transfer test performance, and there was also an interaction between them. Specifically, the disfluent text led to better learning outcomes with or without signaling; however, in the fluent text condition, only signaling facilitated learning. Eye movement analyses indicated that signaling guided learners to pay more attention to important content in the learning materials. The current results suggest that signaling can enhance individuals’ perceived fluency or familiarity to the material and guide the attention during multimedia learning, and the positive impact of disfluency on multimedia learning seems to be more stable and ubiquitous. We discuss these under the framework of disfluency effect and attention-guiding effect.

## Introduction

Multimedia learning refers to the presentation of words and pictures that are intended to foster learning (Mayer, 2017). The words can be in spoken form (e.g., narration) or printed (e.g., on-screen text). The pictures can be static (e.g., illustrations, diagrams, or photos) or dynamic (e.g., animation or video). With the rapid development of science and technology, multimedia teaching has been disseminated widely and applied in both education and other fields. However, multimedia learning materials often contain a substantial amount of information and complex elements. Unreasonable designs may lead to counterproductive effects; for example, information irrelevant to the content may distract learners and impair the learning effect (Lowe, 2003). How to correctly guide learners’ attention and make them focus on the most important content are core elements in promoting the positive effects of multimedia learning.

Perceptual fluency is generally defined as the subjective experience of ease or speed in processing perceptual information. It is usually regarded as a metacognitive experience (Oppenheimer and Frank, 2008). Studies have found that by manipulating the font, font size, color, and clarity of learning materials, learners’ subjective perception of materials difficulty will be increased, resulting in more invested mental effort and analytical processing and ultimately improving academic performance. This finding is called the “disfluency effect” (Eitel et al., 2014). For example, compared to clear fonts and printed images, fuzzy fonts, images, and other fluency interventions have been shown to improve performance (Lee, 2013; Weltman and Eakin, 2014). At present, there are two opposite views on how perceptual disfluency affects learning effect, namely, facilitation and hindering. The theoretical basis of facilitation theory is disfluency theory and desirable difficulty theory. The theoretical basis of the hindrance view mainly involves cognitive load theory (CLT; Xie et al., 2016).

Cognitive load theory argues that the extra cognitive burden stimulated by perceptual disfluency hinders learning. According to the traditional conception of CLT (Sweller et al., 1998), three sources impose a load on working memory when learning through multimedia instruction: internal cognitive load (ICL), extraneous cognitive load (ECL), and germane cognitive load (GCL). ICL is conceptualized as the load on working memory that depends on the interactivity element of the instructional material, as well as the learner’s level of expertise (or prior knowledge). The more complex the material and lower the learner’s level of previous knowledge, the higher the ICL and worse the learning effect. ECL is mainly related to the design of the learning materials or instruction. The worse the design, the higher the ECL, and poorer the learning effect. GCL is the cognitive process related to the learning task itself and is mainly related to the construction of schemas. Unlike ICL and ECL, the higher the GCL, the better the learning effect. Some researchers believe that perceptual disfluency will lead to an increase in ECL (Eitel et al., 2014). Therefore, perceptually disfluent manipulation may be an unproductive way of teaching design in the education process. In other words, after fuzzifying the learning materials, learners need to invest relatively more cognitive resources to deal with the resulting perceptual difficulties increasing the cognitive load. This is why it is not conducive to the learner’s learning effect.

Contrary to the theory of impediments to disfluency, it is believed that the extra cognitive burden stimulated by disfluency promotes learning. Disfluency theory is based on dual-system processing theory. Dual-system processing theory argues that humans have two independent processing systems: System 1, which leads to quick, effortless, more associative and intuitive processing, and System 2, which leads to slow and effortful, more analytic and deliberate processing (James, 1950). Alter et al. (2007) argued that if information processing is perceived as easy (fluent font), it is more likely to activates System 1. If, on the other hand, information processing is perceived as difficult (disfluent font), System 2 will be more likely to be activated, resulting in more invested mental effort and analytical processing. Thus, increasing the perceived difficulty associated with a cognitive task (i.e., disfluency) stimulates deeper processing and more analytical and elaborative thinking. For example, Eitel and Kühl (2016) found that when the fonts of learning materials were relatively difficult to read, learners realized that they had not mastered the materials at the metacognitive level, so the perceived difficulty increased. This type of subjective experience hinders System 1 and activates System 2, forcing learners to invest in more refined analytical thinking and process information more deeply, abstractly, and carefully (Alter et al., 2013), thereby improving the learning effect.

Desirable difficulty theory argues that memory in the learning process includes both storage and retrieval strength. Storage strength refers to the depth of memory of learning content, and retrieval strength references difficulties in extracting content from memory (Bjork and Bjork, 1992). According to desirable difficulty theory (Bjork, 1994), storage and retrieval strength are negatively correlated. That is, the content of low retrieval strength (i.e., information that is harder to extract) increases the length of time that the memory is retained; thus, the learning effect is improved. Perceptually disfluency is a kind of “desirable difficulty” (Katzir et al., 2013), which improves the learner’s storage strength. When the extraction ability is relatively reduced, the learner needs to invest more subjective effort, resulting in a corresponding increase in cognitive burden; thus, the processing of the materials is deeper and the memory maintained longer (Lehmann et al., 2016). For example, Weltman and Eakin (2014) asked learners to perform a five-point Likert self-evaluation of their mastery after studying and found that predicting learning effects based on the perceptual fluency of the materials can easily lead to misjudgment. That is to say, learners believe that fluent material is easier to master than disfluent material, but the latter leads to higher test scores because it provides learners with “desirable difficulty.”

Metacognition is the process of monitoring cognitive activities. The judgment of learning (JOL) is an important form of metacognition monitoring and refers to judgments of the recall possibilities for memory tests after the study phase (Dunlosky and Metcalfe, 2009). JOL significantly affects the subsequent learning processes; for example, people rely on JOL to adjust the time allocation for subsequent study and select the items of study. Researchers have mainly used JOL tasks to investigate the relationship between perceptual fluency and metacognitive judgments in the learning process. For example, Ball et al. (2014) and Rhodes and Castel (2008) argued that fluent materials were likely to cause learners to produce metacognitive illusions during the learning process, leading to overconfidence that in turn would to a certain extent lead to incorrect decision-making. On the contrary, perceptually disfluent learning materials lead to more cautious JOL (Carpenter et al., 2013), which may help alleviate the phenomenon of “overconfidence.” Therefore, perceptual fluency is an important factor that affects learners’ JOL and level of accuracy (Chen et al., 2019). That is, disfluent materials will produce cautious JOL, and fluent materials will produce higher JOL. Chen and Fu (2004) found individuals use different cues to predict their future recall performance. Perceptual characteristics cues, such as brightness (Busey et al., 2000), font size (Hu et al., 2016), text clarity (Pan et al., 2021), volume (Foster and Sahakyan, 2012), and weight (Alban and Kelley, 2013) all affect learning and self-confidence judgments, but not all cues can effectively help learners make accurate JOL (Koriat, 1997). Rhodes and Castel (2008) first revealed the influence of perceptual feature cues on JOL. In their experiment, the perceptual fluency of words in the larger font was significantly better than in the smaller font. Although memory scores of large and small font items were the same, participants mistakenly believed that they were more likely to successfully memorize items in larger font. This phenomenon is called the font-size effect. Zhao et al. (2020) found that as the encoding strength increased, the illusion of JOL caused by font size gradually disappeared. Undorf et al. (2018), Undorf and Bröder (2019), and Hertzog et al. (2013) argued that people will integrate multiple cues when making JOL, but there is no guarantee that individuals will use all given cues. That is, the role of some cues may be ignored by the individual or masked by other cues. Therefore, effective cues are very important. Only when the cues are perceived by learners and bring about a metacognitive experience can they invest more mental effort in the learning material and improve the learning effect. In addition, in previous studies on perceptual fluency, researchers have tended to process all materials (such as the entire text) without fluency. In fact, in a complex learning environment, searching for and absorbing the most important content may be the key to efficient learning (Xie et al., 2016). Therefore, highlighting the important content in learning materials will help learners visually search for that content and optimize memory and understanding (Jamet, 2014; Scheiter and Eitel, 2015).

Signaling is an instructional design method that uses non-content information (such as colors, arrows, and flashing) in multimedia learning to attract learners’ attention, guiding them to focus on key information and promoting learning effects (De Koning et al., 2007, 2010). The positive effect of signaling that guides attention and promotes learning is called the signaling effect (Schneider et al., 2017). A meta-analysis has shown that the signaling effect has good stability (Alpizar et al., 2020). Studies have found that the main reason why signaling guides attention and improves learning effects is that as a prominent stimulus, it can lead learners to pay attention to important information, free up working memory for integrating information and constructing mental representations, and simplify the visual search process to reduce the incorporation of irrelevant information (Kalyuga et al., 1999; Tabbers et al., 2004). However, the attention-guiding hypothesis of signaling has only been verified under specific signaling conditions, such as color (Ozcelik et al., 2010) and contrast changes (De Koning et al., 2007, 2010). Therefore, this study used color signaling to guide learners to pay attention to important content in the learning materials. Because eye movement technology can instantly reflect learners’ cognitive processing and the deeper the learner’s processing the greater the total fixation time, a count of fixations and number of entrances and exits were used as indicators to measure the mechanism of signaling.

In multimedia learning, fluency coding can cause learners to overestimate their mastery of learning materials, resulting in metacognitive bias. Disfluent manipulation can reduce learners’ metacognitive bias, stimulate System 2 to initiate analytical thinking, and encourage learners to invest more mental effort in processing learning materials, thus improving the learning effect. However, there are drawbacks to the facilitation and hindrance theories of disfluency. For example, some scholars have found that there is no difference between fluent and disfluent materials (Ball et al., 2014). However, the current body of research on this topic lacks a systematic and clear explanation. It is believed that this issue can be explored by investigating differences in individual learning of multimedia materials under various processing depths and levels of perceptual fluency. In multimedia learning, this occurs by manipulating the degree of cognitive processing by providing color signaling and then examining how processing depth and perceptual fluency affect each other (in terms of both facilitating and inhibiting learning). As a kind of metacognitive experience, disfluency helps to alleviate cognitive bias in learning, and the attention-guiding effect of signaling can enable individuals to deeply process multimedia materials. In summary, based on disfluency theory and the hypothesis of attention-guiding, we anticipated that disfluency or signaling group may significantly outperform the fluency or non-signaling group in retention and transfer tasks, and there can observe a significant interaction effect between variables of signaling and fluency. We also expected that participants in the signaling group would show more fixation time and counts on AOI than non-signaling group.

## Materials and Methods

### Participants and Design

The experiment recruited 69 participants who met the experimental requirements from Fujian Normal University, China, concerning comparisons based on eye-tracking data, five participants were excluded due to poor eye-tracking data quality (sampling rate less than 60%). The mean age was 20.61years (*SD*=1.88), and there were 47 females and 17 males. Sixteen participants were randomly assigned to each of the four groups derived from a 2×2 between-subjects design with signaling (signaling vs. non-signaling) and fluency (disfluent vs. fluent) as factors. G*Power 3.1 was used to calculate the numbers of participants with a large effect size of *f*=0.40 with power set at 0.80 and alpha set at 0.05 (Faul et al., 2009). The recommended minimum sample size was 52 participants. All participants had normal or corrected-to-normal vision and had to have a prior knowledge score of 60% or less. The main dependent measures were eye-tracking measures and learning outcome post-test scores.

### Materials

#### The Prior Knowledge Test

The test about prior knowledge of meteorology was taken from Moreno and Mayer (1999), which has seven items and employs a five-point Likert scale ranging from very little (1) to very much (5): (a) “I regularly read the weather maps in the newspaper,” (b) “I know what a cold front is,” (c) “I can distinguish between cumulous and nimbus clouds,” (d) “I know what a low-pressure system is,” (e) “I can explain what makes wind blow,” (f) “I know what this symbol means” (symbol for warm front), and (g) “I know what this symbol means” (symbol for cold front).

#### The Subjective Rating Questionnaire

It contains items that assess perceptual fluency, mental effort, cognitive load, and JOL. The perceptual fluency was assessed by “What do you think of the clarity of the text in the material?” on a seven-point scale ranging from 1 (fluent) to 7 (disfluent). Mental effort was assessed by “how much mental effort did you invest?” (Paas and Van Merriënboer, 1993), cognitive load was assessed by “how difficult was it for you to learn with the given material” (Cierniak et al., 2009), and each item had to be rated on a nine-point Likert scale. And the JOL was evaluated by “Please predict the probability that you can answer what you have learned in the subsequent test, the score ranges from 0 to 100% (0% means completely unable to answer, 100% means completely able to answer”).

#### The Retention Test

It comprises one question (Moreno and Mayer, 1999): “Please write down in detail how lightning is formed.” And the participants’ score was computed by counting the number of major idea units (out of 19 possibles).

#### The Transfer Test

It consists of the following four questions: “How can the intensity of lightning be reduced?”; “Suppose you see clouds in the sky and there is no lightning. Why”; “What does air temperature have to do with lightning?”; and “What causes lightning?” According to Moreno and Mayer (1999), we computed a transfer score for each participant by counting the number of acceptable answers that the participant produced across the four transfer problems. Each question contained two acceptable answers, for example, acceptable answers for the first question included removing positive ions from the ground and reducing the temperature difference between the ocean and earth.

#### The Instructional Materials

**Figure 1:**
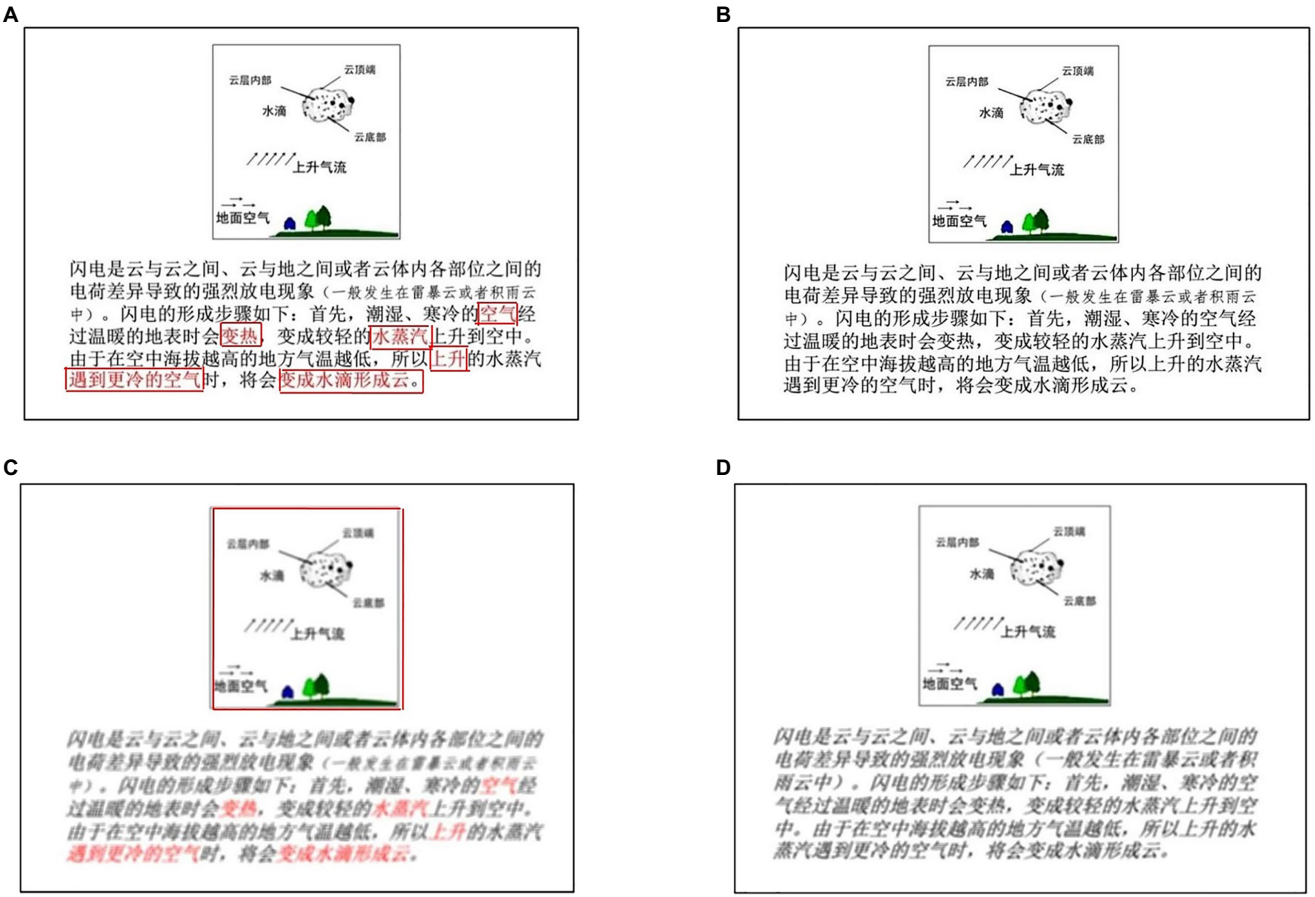
The instructional materials for four experimental conditions. Specifically, **(A)** is the experimental conditions of signaling/fluency, **(B)** is non-signaling/fluency, **(C)** is signaling/disfluency, and **(D)** is non-signaling/disfluency. And the red box on the upper left is the cue interest area, and the lower left is the interest area of the image area. There is no red box of interest areas in the actual experiment process.

The material concerned how lightning develops was adapted from Harp and Mayer (1997) and shown in Figure 1. It was made by a five-page PowerPoint. The disfluent materials were assessed by the university students in advance. Evaluation indicators included “whether the text can be recognized,” “the difficulty of recognizing the text,” “whether the rough operation affects the understanding of the original text,” and “how you think the font is designed.” The material being assessed met the definition of disfluent operability.

### Apparatus

Eye movements were recorded with an EyeLink 1000 Plus Desktop Eye Tracker (SR Research, Canada) with a 1,000Hz sample rate. The experimental materials were presented on a 21-inch monitor with a resolution of 1,280×1,024. The distance between each participant’s eyes and the screen was approximately 65cm. A chin rest was used to minimize head movement.

### Procedure

At first, participants were asked to complete a test about knowledge of meteorology. Then, the participants were told that they will learn an article about how lightning is formed, which consists of five pages. Each page will last 90s and will automatically turn to the next page. They cannot return to read it when time is over. Therefore, they should focus on reading and try their best to understand every step of lightning formation. After the learning phase, students were asked to complete the subjective rating questionnaire to measure their perceptual fluency, mental effort, cognitive load, and the JOL they experienced during learning. Thereafter, they have 15min to complete tests of retention (6min) and transfer (9min).

We also used eye-tracking system to record eye movement data during the reading process. So, after the prior knowledge test, participants were told that they will attend an eye movement calibration test. During calibration, they will be asked to stare at the red dot on the screen until it disappears and to keep their head as still as they can throughout the experiment.

## Results

### Prior Knowledge and Materials Evaluation

Because previous studies have demonstrated that the disfluency effect and signaling effect were stronger for low-experience learners than for high-experience learners, we included only low-experience students in our study. On the pre-test knowledge questionnaire, the scale we used to measure participants’ prior knowledge is a five-point Likert type (from 1 to 5). We set the screening criteria as mean rating across seven items lower than 3, indicating that they have a low-experience in meteorology (Mayer and Moreno, 1998; Moreno and Mayer, 1999). Participants whose mean rating score did not meet the criteria did not attend the subsequent experiment. The mean rating score of all participants was 2.25. A two-factorial ANOVA with signaling and fluency as independent variables and score in the clarity level of the materials evaluation as the dependent variable was conducted, there was a main effect of fluency [*F*(1,60)=161.378, *p*<0.001, η*_p_*^2^=0.729], and the clarity score of the fluent group participants (*M*=6.469, *SD*=0.76) was significantly higher than that of the disfluent group (*M*=3.375, *SD*=1.129). Because the subjective rating scale for disfluency used a seven-point scoring mechanism (1=fluency and 7=disfluency), manipulation of the disfluent materials met the definition of disfluent operability and is consistent with the classification of fluency.

### Learning Outcomes

Two-factorial ANOVAs with signaling and fluency as independent variables and scores for mental effort, materials’ difficulty, JOL, retention, and transfer performance as the dependent variable were conducted (see Table 1). On the score of mental effort, there was neither a main effect of signaling [*F*(1,60)=0.167, *p*>0.05], nor of fluency [*F*(1,60)=0.669, *p*>0.05]. But a significant interaction was observed [*F*(1,60)=6.017, *p*<0.05, η*_p_*^2^=0.091]. A simple effect analysis revealed that under the condition of signaling, the fluency group’s mental effort was significantly higher than that of the disfluency group [*F*(1,60)=5.348, *p*=0.024, η*_p_*^2^=0.082]; in the case of non-signaling, there was no significant difference between the fluency group and the disfluency group [*F*(1,60)=1.337, *p*=0.252]. The average mental effort of the four groups was greater than seven points, indicating that the mental effort of the four groups was high. In terms of the materials’ difficulty, there was neither a main effect of signaling [*F*(1,60)=0.060, *p*>0.05], nor a main effect for fluency [*F*(1,60)=0.329, *p*>0.05], nor an interaction [*F*(1,60)=0.060, *p*>0.05]. Concerning JOL, the main effect of signaling [*F*(1,60)=0.298, *p*>0.05] and fluency [*F*(1,60)=0.019, *p*>0.05], and the interactions of signaling and fluency [*F*(1,60)=0.911, *p*>0.05] were all non-significant. The average value of the task difficulty for the four groups was between 5 and 6, indicating that the learning content was moderately difficult.

**Table 1:** Means (and SD/SE) as a function of text disfluency and signaling.

Variables	Signaling		Non-signaling	
	Fluency	Disfluency	Fluency	Disfluency
Mental effort	7.69(0.95)	6.69(1.66)	7.06(1.06)	7.56(1.09)
Material difficulty	5.44(1.67)	5.75(1.34)	5.63(1.31)	5.75(1.73)
JOL (%)	55.00(18.62)	58.75(19.28)	61.88(13.77)	56.88(20.89)
Retention	7.53(2.51)	8.25(3.17)	8.69(2.93)	9.41(3.36)
Transfer	2.72(1.09)	2.69(0.79)	1.50(1.15)	2.66(1.17)

**Figure 2:**
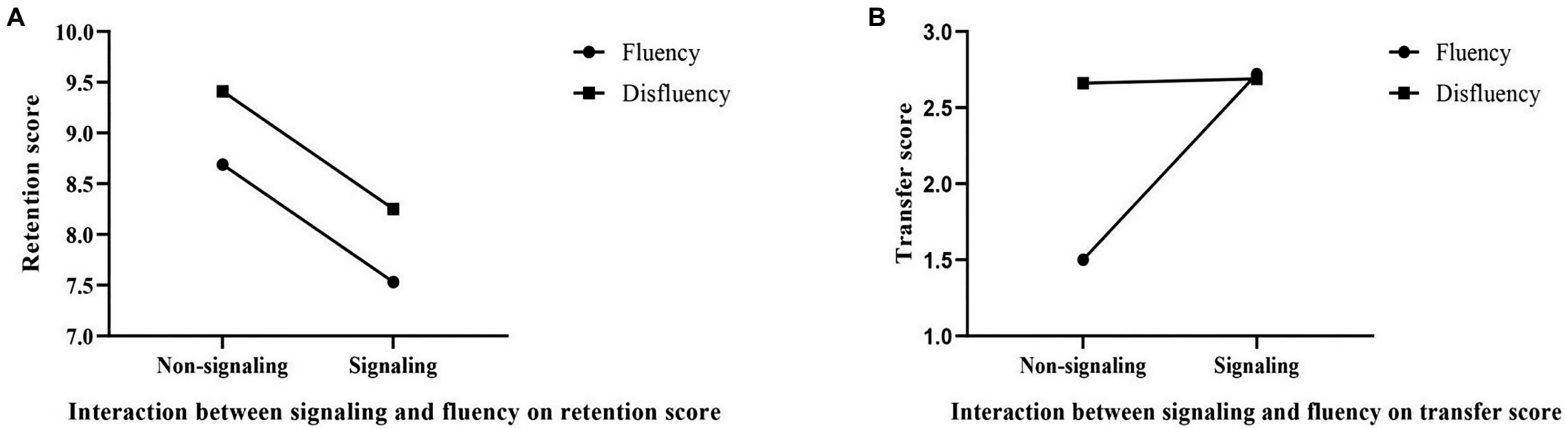
**(A,B)** Interaction between signaling and fluency on retention and transfer score.

Concerning retention, there was neither a main effect for signaling [*F*(1,60)=2.360, *p*=0.130], nor a main effect for fluency [*F*(1,60)=0.912, *p*>0.05], nor an interaction [*F*(1,60)<1, *p*>0.05]. Concerning transfer, a 2×2 ANOVA revealed a main effect of signaling [*F*(1,60)=5.533, *p*=0.022, η*_p_*^2^=0.084], with learners studying with signaling outperforming those with non-signaling. There was a main effect for fluency [*F*(1,60)=4.481, *p*=0.038, η*_p_*^2^=0.069], with learners studying with disfluency outperforming those with fluency. There also revealed an interaction between signaling and fluency [*F*(1,60)=4.993, *p*=0.029, η*_p_*^2^=0.077], and a simple effect analysis revealed that for the fluent condition, the transfer score of the signaling group was significantly higher than that of the non-signaling group [*F*(1,60)=10.519, *p*=0.002, η*_p_*^2^=0.149]; for the disfluent condition, there was no significant difference in transfer performance [*F*(1,60)=0.007, *p*=0.934]. The interaction between signaling and fluency is shown in Figure 2.

Nelson (1984) argued that calculating correlation coefficients such as Pearson’s *r* or a contingency coefficient such as Gamma between JOL and performance scores could be used as a way to measure the relative accuracy and confidence of JOL. The results show that under disfluent conditions, there was a significant positive correlation between JOL and retention score (*r*=0.374, *p*=0.018) and low correlation with transfer score (*r*=0.053, *p*=0.723); under fluent conditions, there was a low correlation between JOL and retention score (*r*=0.248, *p*=0.065) and a significant negative correlation with transfer score (*r*=−0.367, *p*=0.022). According to Schraw (2009), Bias assesses the degree to which an individual is over-or under-confidence when making a confidence judgment. The direction of the discrepancy between confidence and performance provides information about over- vs. under-confidence. The results show that under fluent conditions, the bias index is 0.161 and 0.431 in retention and transfer score, while under disfluent conditions; the bias index is 0.073 and 0.236. We performed independent sample *t*-test for participants’ bias index in fluent and disfluent conditions, the result shows there is no significant difference on retention scores [*t*(30)=1.384, *p*=0.176], but there is a significant difference on transfer score [*t*(30)=2.441, *p*=0.021], and the bias index of the fluent condition (*M*=0.431, *SD*=0.214) was significantly higher than that of the disfluent condition (*M*=0.237, *SD*=0.237).

### Outcomes of Eye-Tracking

To explore whether an improvement in learners’ academic performance could be caused by processing more pictures, the interest areas of particular pictures were analyzed, and the results are shown in Table 2. Due to there being different areas in the images and text of the material, the proportion of fixation time on the picture area (referring to the ratio of total fixation time on the picture area to total fixation time on the entire set of materials) and proportion of the fixation count for the picture area (referring to the ratio of the number of fixation points in the picture area to the number of fixation points in the entire set of materials) were used as indicators of the distribution of graphic attention. The number of transitions between the text and picture area of interest (Run Count of AOI refers to the number of times the participant looked back and forth between the picture and other areas; the higher the number, the more attention that was paid to the area of interest) was used as an indicator of the level of integration of the picture and text (Alemdag and Cagiltay, 2018). With regard to the proportion of fixation time on the image area of interest, a 2×2 ANOVA revealed neither a main effect for signaling [*F*(1,60)=1.040, *p*>0.05], nor a main effect for fluency [*F*(1,60)=0.006, *p*>0.05], nor an interaction [*F*(1,60)=0.051, *p*>0.05]. With regard to the proportion of the count of fixations on the picture area, a 2×2 ANOVA showed the main effect of signaling [*F*(1,60)=1.162, *p*>0.05] and fluency [*F*(1,60)=0.256, *p*>0.05], and the interaction of signaling and fluency [*F*(1,60)=0.371, *p*>0.05] were all non-significant. With regard to the number of transitions between the text and picture area of interest, a 2×2 ANOVA showed neither a main effect for signaling [*F*(1,60)=0.221, *p*>0.05], nor a main effect for fluency [*F*(1,60)=0.501, *p*>0.05], nor an interaction [*F*(1,60)=0.515, *p*>0.05].

**Table 2:** Means and SDs for eye-tracking measures.

AOI	Eye movement indicator	Signaling		Non-signaling	
		Fluency	Disfluency	Fluency	Disfluency
Picture	Proportion of fixation time	0.21(0.08)	0.21(0.08)	0.19(0.09)	0.19(0.06)
	Proportion of fixation count	0.20(0.07)	0.18(0.07)	0.17(0.07)	0.17(0.05)
	Run count	54.44(25.84)	54.50(20.40)	55.94(26.71)	47.31(23.38)
Signaling	Fixation time (s)	62.84(11.21)	66.63(11.22)	58.56(13.32)	55.89(10.39)
	Fixation count	269.63(34.66)	290.75(57.53)	246.81(44.70)	236.19(37.40)
	Run count	180.88(18.82)	197.00(34.38)	154.63(23.00)	158.63(24.93)

To explore the influence mechanism of signaling on learning results, this study considered the area of the signaling in the material as the area of interest and compared the differences in eye movement among different experimental groups, the results are shown in Table 2. The selected eye movement index used the fixation time of AOI (that is, the time that a participant’s eyes stopped in the area of interest during the learning process), count of fixations (referring to the number of fixation points in the text area of the subject’s gaze; the greater the value, the higher the number of fixations), and the number of transitions between signaling and non-signaling areas of interest (referring to the number of times the participant looked back and forth between the cue and other areas; The higher the value, the more attention paid to the area of interest). With respect to the fixation time, there was a main effect of signaling [*F*(1,60)=5.674, *p*=0.020, η*_p_*^2^=0.086], with learners studying the signaling gaze longer than did the non-signaling group, but there was no main effect for fluency [*F*(1,60)=0.031, *p*>0.05], and no interaction [*F*(1,60)=1.048, *p*>0.05]. Concerning count of fixations, results revealed a significant main effect of signaling [*F*(1,60)=12.115, *p*=0.001,η*_p_*^2^=0.168], with learners studying the signaling gaze longer than did the non-signaling group. Moreover, there was no main effect of fluency [*F*(1,60)=0.223, *p*>0.05], and no interaction [*F*(1,60)=2.040, *p*>0.05]. Concerning the number of transitions between signaling and non-signaling areas of interest, there was a significant main effect of signaling [*F*(1,60)=24.872, *p*<0.001, η*_p_*^2^=0.293], with learners studying under the signaling condition having more transitions between text and picture than did the non-signaling group. But the main effect of fluency [*F*(1,60)=2.412, *p*>0.05] and the interaction of signaling and fluency [*F*(1,60)=0.876, *p*>0.05] were all non-significant.

## Discussion

This research investigated the impact of perceptual fluency and signaling on the processing of multimedia learning graphics and explored the role of color signaling in multimedia learning. The results show that the transfer scores for the disfluency group were significantly higher than for the fluency group, and the scores for the signaling group were significantly higher than those for the non-signaling group. Under fluent conditions, the transfer performance of the signaling group was significantly higher than that of the non-signaling group, while under disfluent conditions, there was no difference.

In multimedia learning, retention tests mainly determine learners’ memorization abilities as reflected by the number of items learned or how much each learner memorizes. The retention test only requested participants recall what they had learned, which may have been affected by each learner’s working memory capacity. Lehmann et al. (2016) believed that the additional demands on working memory made by disfluent texts may only apply to learners with high levels of working memory capacity, and only learners with such high levels will have better retention performance under disfluent conditions. Therefore, regardless of whether the material is fluent or not, the learning effect is the same. The results show that there was no significant difference in retention performance between the fluency and disfluency groups, which is not completely consistent with previous research findings. Both the desirable difficulty and disfluency theories argue that disfluent material is beneficial to the improvement of learning effects; while CLT takes the position that the extra cognitive load stimulated by perceptual fluency hinders learning. Other experimental studies have found that disfluency operation does not affect learning. The reason why there is no significant difference in learning effects may be the result of boundary conditions. For example, the disfluency object, manipulation method, and degree of manipulation may affect the disfluency effect. However, the experimental materials used in this study were evaluated in advance. The positions of pictures and text, font size, and prior knowledge of participants were also all controlled. Therefore, this research found that the reason for the lack of fluency was due to the boundary condition of familiarity; that is to say, learners may at first perceive materials in a certain font as disfluent, but over time, they may come to perceive them as fluent, due to an increase in familiarity (French et al., 2013; Lehmann et al., 2016). Therefore, individuals first reduce mental effort when encountering a disfluent text, and then increase their mental effort after a period of time (Strukelj et al., 2016). The results of the present study show that the four groups of subjects all put in more mental effort when studying.

In multimedia learning, the transfer score reflects the learner’s ability to understand the learning materials and use them in new situations and indicates the quality of the learning. This study found that the transfer score of the disfluency group was significantly higher than that of the fluency group, which is consistent with previous research (Eitel et al., 2014) and indicates that disfluent texts make learners find it more difficult to read on a perceptual level, perhaps cuing them to process more deeply and stimulate their analytical thinking, resulting in their putting in more mental effort. We used pictures in the material as an area of interest, finding that the effect of fluency was not significant with regard to eye movement indicators such as the proportion of the time of fixation and number of transitions to the area of interest. This eliminates the possibility that learners learn through image integration to obtain better test scores. It also proves that in multimedia learning, learners show a text-orientation in their reading.

There was no significant difference in retention performance between the signaling and non-signaling groups, but there was a significant difference in the transfer performance. The transfer performance of the signaling group was significantly higher than that of the non-signaling group, indicating that color signaling had an impact on deeper processing, and reasonable inclusion of signaling in learning materials can enhance the perceptual salience of key information. In other words, color signaling has an attention-guiding effect, and it can maintain learner’s attention on that information, causing them to invest more attention resources on it and promoting learning effects; this is consistent with previous studies (Mautone and Mayer, 2001; Ozcelik et al., 2010). In terms of eye movement indicators, this study also found that in the signaling areas of interest, the fixation time, count of fixations, and the number of transitions between signaling and non-signaling areas of interest were significantly higher than for non-signaling areas. Thus, the attention-guiding effect of signaling can make the individual faster searching for important areas of learning content, focus on specific key areas, and ignore areas unrelated to the task, thereby improving the learning effect.

This research also determined that the interaction between signaling and fluency was significant in terms of transfer scores, and color signaling being added to a disfluent text will not significantly increase or decrease test scores. This study also found that adding color signaling to a fluent text would improve test scores. Disfluency theory argues that disfluent texts manipulate the surface complexity of the material. When the learning material becomes blurred, the learner’s “perceived difficulty” increases, which reduces the cognitive bias of learning caused by fluency and thereby alleviates overconfidence. At the same time, disfluency manipulation activates analytical thinking of System 2 and makes learners invest more mental effort on processing information and improving their understanding of learning materials. Therefore, regardless of whether there is a signaling condition, disfluent manipulation makes learners experience “perceptual difficulty” at a metacognitive level, stimulates System 2, and activates analytical thinking. That is, regardless of the conditions, disfluency manipulation promotes improvement in test scores. This reflects the stability and universality of the disfluency effect in the multimedia learning framework. Under fluency conditions, learners do not perceive the difficulty caused by characteristics external to the material at the metacognitive level, so System 2 will not be stimulated to activate analytical thinking. Thus, under the condition of non-signaling, performance is poor. Under the signaling condition, although fluent material fails to stimulate System 2 and initiate analytical thinking, signaling has an attention-guiding effect in multimedia learning that can guide learners to pay attention to related information and simplify their visual search process. From another angle to the interaction, we found that signaling can improve learning effect whether the text is fluent or not, it also reflected the stability of the signaling effect, and combining with the eye movement indicators, we conclude that signaling can guide the learners’ attention and enhance individual’ perceived fluency and familiarity to the material. Thus, it can help learners find important information, release working memory to integrate information and construct mental representations.

Results about JOLs showed that, learners’ JOL and test scores were significantly positively correlated under disfluent conditions, which suggests that disfluent operation may improve the accuracy of participants’ JOL. According to the result of the bias index, we found there is a significant difference in transfer score, and the bias index of the fluent condition was significantly higher than that of the disfluent condition, indicating that perceptually fluent learning materials are prone to producing metacognitive illusions (over-confidence) during the learning process. Although the data showed that disfluent manipulation alleviated the learner’s over-confidence, we only observed a significant difference in transfer (not retention) performance test. We speculated that this might be because the retention test mainly examined the verbatim remembering ability which is relatively less susceptible to metacognition; however, the transfer test reflects the learner’s ability to understand the material and apply what they learned to solve problems which may be greatly influenced by over-confidence illusion (e.g., give up too early to achieve deep learning; BjorK and Kroll, 2015). These results are consistent with the research hypotheses.

Under the conditions of this research, the following conclusions can be drawn. Firstly, in multimedia learning, signaling can effectively guide learners’ attention and promote depth of learning and materials processing; this is supported by eye movement research, and signaling also can enhance individuals’ perceived fluency or familiarity to the learning materials. Therefore, in the teaching process, educators can use color signaling to highlight important knowledge points when presenting teaching content with complex elements. Secondly, metacognition caused by experiencing disfluency can reduce learners’ cognitive bias and may become a cue for learners to process more deeply. This work also illustrates the stability and universality of the positive impact of disfluency on multimedia learning. Although it seems unreasonable to make the text *slightly harder-to-read* (i.e., disfluent) in the teaching practice, we can get some inspiration based on this research. For example, set up questions with desirable difficulty for students to make them involve in mental effort during learning.

Some limitations of this study should be acknowledged. First, this study verified that mental effort benefits transfer learning; however, based on the current data, we barely know the underlying mechanism. The eye movement only revealed learners’ attention pattern between the signaled text and unsignaled text, and most of the discussions were inferred from behavioral data. Second, the transfer tasks employed in this study were more of a near transfer, extend them to far transfers will promote educational and practical implications of the research.

## Data Availability Statement

The original contributions presented in the study are included in the article/Supplementary Material; further inquiries can be directed to the corresponding author.

## Ethics Statement

The studies involving human participants were reviewed and approved by Fujian Normal University. The patients/participants provided their written informed consent to participate in this study.

## Author Contributions

TL and JZ developed the study concept and contributed to the study design. Testing and data collection were performed by TL. TL performed the data analysis and interpretation under the supervision of JZ, and TL drafted the manuscript, and JZ provided critical revisions. All authors contributed to the article and approved the submitted version.

## Funding

This work was supported by the Key Research Institute of Humanities and Social Sciences, Ministry of Education, China (Grants 16JJD190004).

## Conflict of Interest

The authors declare that the research was conducted in the absence of any commercial or financial relationships that could be construed as a potential conflict of interest.

## Publisher’s Note

All claims expressed in this article are solely those of the authors and do not necessarily represent those of their affiliated organizations, or those of the publisher, the editors and the reviewers. Any product that may be evaluated in this article, or claim that may be made by its manufacturer, is not guaranteed or endorsed by the publisher.

## Supplementary Material

Supplementary Table S1 The experimental materials about the prior knowledge test and the subjective rating questionnaire.
