# Supplementary material for: The Influence of Signaling on the Disfluency Effect in Multimedia Learning
Source: Front Psychol. 2021 Nov 2;12:755804. doi: 10.3389/fpsyg.2021.755804 (PMC8593463; doi:10.3389/fpsyg.2021.755804)
Supplement: Supplementary Table S1 — The experimental materials about the prior knowledge test and the subjective rating questionnaire. [file Table_1.DOCX]

**Supplementary Material**

**The pre-test questionnaire**

**
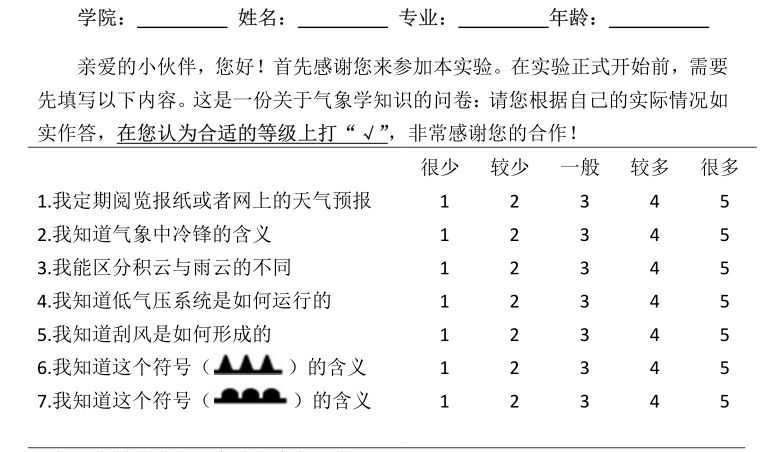
**

**The subjective rating questionnaire**

**
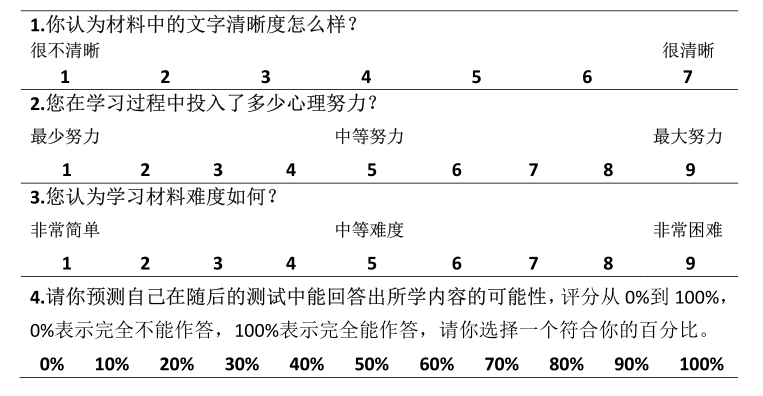
**

**The retention test**

**1.请你尽可能详细地写出闪电形成的基本过程。**

**The transfer test**

**2.如何才能降低闪电的强度?**

**3.假设你看见天空中的云层却没有出现闪电，这是为什么?**

**4.气温与闪电有什么关系?**

**5.什么导致了闪电的产生?**
